# Revisiting the association between maternal and offspring preterm birth using a sibling design

**DOI:** 10.1186/s12884-019-2304-9

**Published:** 2019-05-29

**Authors:** Marcelo L. Urquia, Elizabeth Wall-Wieler, Chelsea A. Ruth, Xiaoqing Liu, Leslie L. Roos

**Affiliations:** 1 Manitoba Centre for Health Policy, Department of Community Health Sciences, Rady Faculty of Health Sciences, University of Manitoba, 408-727 McDermot Avenue, Winnipeg, Manitoba R3E 3P5 Canada; 2 Departments of Obstetrics, Gynecology and Reproductive Sciences; Biochemistry and Medical Genetics, Rady Faculty of Health Sciences, University of Manitoba, Winnipeg, Manitoba Canada

**Keywords:** Preterm birth, Preterm delivery, Recurrence, Family, Siblings, Sisters, Cousins, Intergenerational, Fixed-effects

## Abstract

**Background:**

Previous studies have reported an intergenerational association between maternal and offspring preterm birth (PTB) but the nature of the association remains unclear. We assessed the association between maternal and offspring preterm birth using a quasi-experimental sibling design and distinguishing between preterm birth types.

**Methods:**

We conducted a retrospective intergenerational cohort study of 39,573 women born singleton in Manitoba, Canada (1980–2002) who gave birth to 79,198 singleton infants (1995–2016). To account for familial confounding we defined a subcohort of 1033 sisters with discordant PTB status who subsequently gave birth and compared offspring PTB rates between 2499 differentially exposed cousins using log-binomial fixed-effects generalized estimating equation models. PTB was defined as a delivery < 37 gestation weeks, divided into spontaneous and provider-initiated.

**Results:**

In the population cohort, mothers born preterm were more likely to give birth preterm [Adjusted Relative Risk (ARR): 1.39; 95% Confidence Interval (CI): 1.25, 1.54] and very preterm birth [ARR: 1.76; 95% CI: 1.29, 2.41]. However, in the siblings cohort, the intergenerational association was not apparent among births to sisters with discordant PTB status [ARR: 1.02; 95% CI: 0.77, 1.34 for preterm birth and ARR: 0.88; 95% CI: 0.38, 2.02 for very preterm birth]. Mothers born at term with a sister born preterm had a similarly elevated risk of delivering a preterm infant (10%) than their preterm sisters. Intergenerational patterns were observed for spontaneous PTB but not for provider-initiated PTB.

**Conclusions:**

Our findings suggest that it is not the fact of having been born preterm that puts women at higher risk of delivering preterm, but the fact of having been born to a mother who ever delivered preterm. Consideration of a female family history of PTB may better identify women at higher risk of preterm delivery than relying on maternal preterm birth status alone. Further research may benefit from distinguishing preterm birth types.

## Background

Preterm birth (PTB) is one main factor associated with infant mortality and morbidity in industrialized countries [1, 2]. Preterm infants, especially those born earliest, are more likely to experience a myriad of health-related problems, from neurodevelopmental problems in childhood to cardiovascular and metabolic disorders later in life [3].

The etiology of preterm birth is multifactorial, complex and not well understood [4]. One important piece in the puzzle is the intergenerational recurrence of PTB. Several studies have consistently shown that mothers born preterm are more likely to have preterm babies [5–8] although the mechanisms behind the association remain unclear. A meta-analysis summarizing unadjusted estimates recommended including maternal birth status in risk assessment when planning pregnancy care for individual women [9]. Meta-analyses of observational studies, however, should be taken with caution since spurious summary results may result from systematic biases shared by the included studies [10]. Although randomized trials are not an option to study this association, previous studies have rarely used observational designs for causal inference, such as sibling designs [11]. Being born preterm may not necessarily be an individual risk factor for delivering preterm infants. Rather, it may merely reflect the persistence of unmeasured environmental and family (genetic and behavioral) risk factors transmitted from one generation to another [12–15]. Quasi-experimental sibling studies are well suited to isolate the causal effect of an individual risk factor not shared by siblings.

Our goal was to assess whether maternal preterm birth status is an individual risk factor per se for delivering preterm. To distinguish the risk of delivering preterm among women born preterm themselves from that of women born at term but with a family history of preterm birth, we used fixed-effects methods to compare preterm birth rates among the offspring (cousins) of sister mothers of discordant preterm birth status. Furthermore, we distinguished between spontaneous and provider-initiated PTB, which may be differentially involved in the recurrence of PTB across generations [16–19].

## Methods

### Design and study population

This retrospective cohort study used linked population-based health and social registers, accessed through the Manitoba Population Research Data Repository. Manitoba is Canada’s central province with an area slightly higher than France and 1.28 million inhabitants by the 2016 Census, 72% of whom live in urban areas. All Manitoba residents are covered by the publicly funded provincial health insurance plan. The study population included all women born singleton in a Manitoba hospital from 1980 to 2002 who subsequently gave birth to a singleton live birth in the province at or after 15 years of age, from 1995 to 2016. Mother-child clusters including multiple births, missing or extreme (< 24 or > 42 weeks) gestational age were excluded.

We used two cohorts to make comparisons. First, to allow comparability with previous studies, we assessed the association among all women giving birth in the general population. Like in most previous studies, PTB rates of children born to mothers who were born preterm were compared to the PTB rates of children born to mothers born at term. Most mothers in this population cohort (71%) did not have a sister giving birth in the study period, which makes it impossible to separate individual from familial influences due to the absence of a within-family comparator, that is, a sister who shares the genetic and family environment but differs in preterm birth status. Secondly, to overcome this limitation, we used a subset of the population cohort composed of sisters of discordant preterm birth status (i.e., at least one sister was born preterm and at least one was not). This sibling subcohort allows for comparison of risk of PTB between the offspring (cousins) of sisters of discordant PTB status while controlling for stable family characteristics shared by sister mothers using fixed-effects analysis [20–22].

### Data sources and variables

We used deterministically linked province-wide health and social databases. Maternal obstetrical and newborn hospital discharge abstracts are cross-referenced (99.6% valid matches) and were used to identify sisters born to the same biological mother, PTB status, date of birth, birth order, maternal age and infant sex. A validation study found the hospital abstracts to be adequately accurate to assess perinatal morbidity [23]. PTB was defined as a clinical estimate of gestational age below 37 completed weeks. Early PTB was defined as a gestational age below 32 weeks and moderately to late PTB from 32 to 36 weeks gestation. Information on gestational age was recorded in the hospital separation abstracts by attending physicians and nurses based on the clinical information collected during the hospitalization, which may include menstrual dates, ultrasonography and newborn examination, although the exact contribution of each is unknown. Spontaneous PTB was assessed by the International Classification of Diseases, Ninth Revision, Clinical Modification (ICD-9-CM) [24], codes 644, 658.1 or 658.2 before April 1, 2004 and after that date by the International Classification of Diseases, Tenth Revision, Canada [25], codes O601, O42 or O756. The remainder of PTB cases were considered provider-initiated.

The other linkable databases in the Manitoba repository are also of good quality and have evolved over time [26, 27]. Information on neighborhood socioeconomic status was obtained from the Canadian Census closest to the infant’s birthdate and aggregated at the dissemination area level, the smallest geographic area for which all census data are reported, typically composed of 400 to 700 persons. Neighborhood socioeconomic level was measured by the Socioeconomic Factor Index [28], which combines neighborhood information on income, education, employment, and family structure and was categorized into tertiles and unknown. Location of neighborhood and residential mobility were measured by residential postal codes available from the provincial Health Insurance Registry, information that accrues longitudinally and is updated semi-annually.

Physician claims for visits in offices, hospitals and outpatient departments are compiled by the provincial Ministry of health in the Permanent Medical Statistical file and were used to assess maternal substance abuse in the year preceding the birth of the child, in combination with hospital records. Substance abuse was defined as one or more physician visits with a diagnosis for alcohol or drug psychoses, alcohol or drug dependence, or nondependent abuse of drugs (ICD-9-CM codes 291, 292, 303, 304, 305). The same codes were used for hospitalizations occurred before April 1, 2004. ICD-10-CA codes F10-F19, F55 were used for hospitalizations occurred on or after April 1, 2004.

### Data analysis

Descriptive frequencies were provided according to maternal sibling patterns. A few variables were re-categorized to prevent small cell counts, which cannot be disclosed according to provincial privacy legislation. Remaining cells with small counts were suppressed in our tables.

To assess the association between maternal and offspring PTB in the population cohort (Tables 2 and 3) we used log-binomial regression to obtain Relative Risks with 95% CI, before and after adjustment for available potential confounding factors.

In the sibling subcohort we conducted a matched cohort analysis (Tables 2 and 4). Each cluster was composed of two or more sister mothers of discordant PTB status who gave birth in the study period. We used fixed-effects log-binomial regression to estimate the association between maternal and child PTB within sister clusters [29]. Generalized Estimating Equations were used to account for the clustering of sister mothers within grandmothers and unequal sister-mothers and offspring cluster sizes. For each approach, we further performed stratified analyses according to PTB type.

In sensitivity analyses restricted to a subset of women giving birth at or after 2000 with complete information on additional potential confounders, we further controlled for individual-level characteristics that may differ between sister-mothers, such as adequacy of prenatal care based on the Revised-Graduated Prenatal Care Utilization Index, smoking during pregnancy, maternal education, single marital status and receipt of employment income assistance. A second sensitivity analysis was restricted to one randomly selected child per mother to account for the recurrence of PTB among different pregnancies of a given woman, which could inflate the numerator and therefore the effect estimates.

## Results

There were 58,628 mothers born on or after April 1st, 1980 who gave birth to 116,790 children from April 1st, 1995 to March 31, 2016. We excluded mother-infant pair records that met one or more of the following exclusion criteria. Missing maternal gestational age (*N* = 17,687), extreme maternal gestational age (*N* = 242), mother born from multiple pregnancy (*N* = 356), missing child’s gestational age (*N* = 4613), child is a multiple (*N* = 2735) and extreme child’s gestational age (*N* = 4824). After exclusion of 19,055 mothers (32.5%) and 37,592 children (32.2%), the population cohort for analyses was composed of 39,573 mothers and 79,198 children. Of these mothers, 28,133 (71.1%) did not have a sister giving birth during the study period.

Among the 11,440 sisters, 1033 (9.0%) had discordant preterm birth status (455 born preterm and 578 at term) (Table 1), 69 (0.6%) were all preterm and the vast majority of sisters (*n* = 10,338; 90.4%) were born at term. Overall, offspring preterm birth rates were 6.2 per 100 live births in the population. Mothers without sisters giving birth in the cohort were more likely to deliver preterm if they themselves were born preterm. Among sister mothers with discordant PTB status, offspring preterm birth rates did not differ (10.2% among sisters born preterm and 10.0% among sisters born at term). The highest PTB rate occurred in a small number of families where all sister mothers were born preterm (13.1%) and the lowest rate where all sister mothers were born at term (6.1%).

**Table 1:** Characteristics of the study population, Manitoba, Canada, 1980-2016^a^

	Population Cohort	Mothers without sisters		Mothers with sisters			
				Discordant sisters		Concordant sisters	
		Mother Born Preterm	Mother Born at term	Index mothers born preterm with sisters born at term	Index mothers born at term with sisters born preterm	All born preterm	All born at term
Number of mothers (%)^b^	39,573 (100.0)	1328 (3.4)	26,805 (67.7)	455 (1.2)	578 (1.5)	69 (0.2)	10,338 (26.1)
Number of infants (%)^b^	79,198 (100.0)	2565 (3.2)	50,647 (63.9)	1069 (1.4)	1430 (1.8)	137 (0.2)	23,350 (29.5)
Infant’s preterm birth	4913 (6.2)	198 (7.7)	3025 (6.0)	109 (10.2)	143 (10.0)	18 (13.1)	1420 (6.1)
Infant’s preterm birth type							
Provider-initiated	1489 (1.9)	44 (1.7)	912 (1.8)	34 (3.2)	53 (3.7)	< 6^c^	443 (1.9)
Spontaneous	3424 (4.3)	154 (6.0)	2113 (4.2)	75 (7.0)	90 (6.3)	c	977 (4.2)
Infant’s Birth Year, mean(SD)	2010 (4.3)	2010 (4.4)	2010 (4.3)	2010 (4.2)	2009 (4.4)	2009 (4.3)	2010 (4.2)
Male infant	40,624 (51.3)	1290 (50.3)	26,037 (51.4)	547 (51.2)	765 (53.5)	70 (51.1)	11,915 (51.0)
Infant’s Birth Order							
1	39,069 (49.3)	1321 (51.5)	26,495 (52.3)	449 (42.0)	567 (39.7)	67 (48.9)	10,170 (43.6)
2	22,505 (28.4)	727 (28.3)	14,559 (28.8)	281 (26.3)	374 (26.1)	42 (30.7)	6522 (27.9)
3	9930 (12.5)	299 (11.7)	5727 (11.3)	165 (15.4)	212 (14.8)	17 (12.4)	3510 (15.0)
4+	7694 (9.7)	218 (8.5)	3866 (7.6)	174 (16.3)	277 (19.4)	11 (8.0)	3148 (13.5)
Mother’s Age							
< 20	17,467 (22.1)	504 (19.6)	10,306 (20.3)	251 (23.5)	422 (29.5)	29 (21.2)	5955 (25.5)
20–24	28,714 (36.3)	915 (35.7)	17,654 (34.9)	438 (41.0)	581 (40.6)	51 (37.2)	9075 (38.9)
25–29	23,741 (30.0)	812 (31.7)	15,856 (31.3)	281 (26.3)	321 (22.5)	38 (27.7)	6433 (27.6)
30 +	9276 (11.7)	334 (13.0)	6831 (13.5)	99 (9.7)	106 (7.4)	19 (13.9)	1887 (8.1)
Neighborhood SES tertiles							
P0-P33 (Lowest SES)	25,940 (32.8)	753 (29.4)	13,341 (26.3)	560 (52.4)	794 (55.5)	c	10,444 (44.7)
P34-P66	25,244 (31.9)	870 (33.9)	16,983 (33.5)	298 (27.9)	373 (26.1)	57 (41.6)	6663 (28.5)
P67-P100 (Highest SES)	25,219 (31.8)	846 (33.0)	18,266 (36.1)	197 (18.4)	240 (16.8)	27 (19.7)	5643 (24.2)
Unknown	2795 (3.5)	96 (3.7)	2057 (4.1)	14 (1.3)	23 (1.5)	< 6 ^c^	600 (2.6)
Location of Neighborhood							
Urban	35,674 (45.0)	1364 (53.2)	25,017 (49.4)	398 (37.2)	514 (35.9)	75 (54.7)	8306 (35.6)
Rural South	17,364 (21.9)	483 (18.8)	11,079 (21.9)	196 (18.3)	246 (17.2)	22 (16.1)	5338 (22.9)
Rural Mid	11,891 (15.0)	362 (14.1)	7378 (14.6)	182 (17.0)	237 (16.6)	22 (16.1)	3710 (15.9)
Rural North	14,269 (18.0)	356 (13.9)	7173 (14.2)	293 (27.4)	433 (30.3)	18 (13.1)	5996 (25.7)
Mother moved in Year before Birth of Child	20,819 (26.3)	728 (28.4)	13,518 (26.7)	295 (27.6)	378 (26.4)	34 (24.8)	5866 (25.1)
Mother had a Substance Abuse Diagnosis in Year before Birth of Child	2111 (2.7)	67 (2.6)	1174 (2.3)	37 (3.5)	67 (4.7)	< 6 ^c^	761 (3.3)
Mother had no partner^d^	6400 (8.52)	220 (8.92)	4120 (8.55)	83 (8.84)	113 (9.28)	11 (9.65)	1853 (8.70)
Mother smoked during pregnancy^d^	19,257 (26.0)	688 (27.91)	11,435 (23.74)	317 (33.76)	438 (35.96)	34 (29.82)	6345 (29.78)
Inadequate prenatal care^d^	13,548 (18.26)	406 (16.47)	7270 (15.09)	271 (28.86)	378 (31.03)	22 (19.30)	5201 (24.41)
Employment income assistance ^d^	15,631 (21.06)	578 (23.45)	9383 (19.48)	334 (27.42)	246 (26.20)	32 (28.07)	5058 (23.74)
Maternal education^d^							
12 years or less	17,692 (23.84)	541 (21.95)	9829 (20.40)	336 (35.78)	481 (39.49)	20 (17.54)	6485 (30.44)
More than 12 years	42,885 (57.79)	1496 (60.69)	30,512 (63.34)	379 (40.36)	426 (34.98)	80 (70.18)	9992 (46.90)
Unknown	13,637 (18.38)	428 (17.36)	7831 (16.26)	224 (23.86)	311 (25.53)	14 (12.28)	4829 (22.66)

Abbreviations: *SD* Standard Deviation, *SES* Socioeconomic Status

^a^Frequencies are expressed as N (column %) unless otherwise specified

^b^Row percent

^c^Suppressed due to small cell size

^d^Information available for births occurred from 2000 to 2016 (Population cohort: 38,212 mothers, 74,214 children; sibling subcohort: 951 mothers, 2157 children)

Infant’s higher birth order was more common among mothers with sisters in the cohort, particularly among discordant sisters, as were other characteristics associated with adverse birth outcomes, such as teenage pregnancy, low socioeconomic status, residing in the rural North (mostly inhabited by Indigenous populations), history of substance abuse, smoking during pregnancy, inadequate prenatal care, receiving income assistance and low maternal education (Table 1). Infants whose mothers or aunts were born preterm had the most adverse profiles, which differed little between discordant sisters.

In the population cohort analysis, infants exposed to maternal preterm birth had higher risk of preterm birth before, and after adjustment [Adjusted Relative Risk (ARR): 1.38; 95% CI: 1.25, 1.55] (Table 2). The association was stronger when the mother’s gestational age was < 32 weeks (ARR: 1.77; 95% CI: 1.28, 2.42). In the sibling analysis, comparing sisters of discordant PTB status, maternal PTB was no longer associated with offspring PTB before or after adjustment overall (ARR: 1.02; 95% CI: 0.77, 1.34) or according to length of gestation.

**Table 2:** Association between maternal and offspring preterm birth, Manitoba, Canada, 1980–2016

Maternal gestational age	Infant’s preterm birth cases/No of births	Preterm birth rate, %	URR	95% CI	ARR ^c^	95% CI
Population cohort^a^ (*N* = 79,198)						
Not Preterm	4588/75,427	6.08	1.00	Reference	1.00	Reference
Preterm (24–36 weeks)	325/3771	8.62	1.42	1.27, 1.58	1.39	1.25, 1.54
Very Preterm (24–31 weeks)	35/326	10.74	1.77	1.28, 2.42	1.76	1.29, 2.41
Moderately Preterm (32–36 weeks)	290/3445	8.43	1.38	1.24, 1.55	1.35	1.21, 1.51
Sibling subcohort^b^ (*N* = 2499)						
Not Preterm	143/1430	10.00	1.00	Reference	1.00	Reference
Preterm (24–36 weeks)	109/1069	10.20	1.02	0.77, 1.36	1.02	0.77, 1.34
Very Preterm (24–31 weeks)	7/80	8.75	0.88	0.39, 1.96	0.88	0.38, 2.02
Moderately Preterm (32–36 weeks)	102/989	10.31	1.03	0.77, 1.38	1.03	0.78, 1.36

Abbreviations: *URR* Unadjusted Relative Risk, *ARR* Adjusted Relative Risk, *CI* confidence intervals

^a^Analyzed with a log-binomial regression model

^b^Analyzed with a GEE fixed-effects log-binomial regression model

^c^Adjusted for whether mother moved or had a substance abuse diagnosis in year before birth of child, child’s birth year, mother’s age at birth of child, neighborhood location and SES at birth of child, child’s sex and child’s birth order

Analyses splitting PTB into types in the whole population indicate that both maternal spontaneous and provider-initiated PTB were associated with spontaneous offspring PTB (Table 3), although maternal provider-initiated PTB was no longer associated with offspring PTB in analyses restricted to one child per mother. Maternal preterm birth types were not associated with offspring provider-initiated PTB before or after adjustment.

**Table 3:** Association between maternal and offspring preterm birth type in the population cohort ^a^, Manitoba, Canada, 1980–2016

		Infant’s PTB cases/No of births	PTB rate, %	(*N* = 78,922)		(*N* = 78,922)		(*N* = 74,196)		(*N* = 39,478)	
Offspring outcome	Maternal PTB type			URR	95% CI	ARR^b^	95% CI	ARR^c^	95% CI	ARR^d^	95% CI
Any PTB	Not Preterm	4565/75,172	6.07	1.00	Reference	1.00	Reference	1.00	Reference	1.00	Reference
	Spontaneous	195/2165	9.01	1.48	1.29, 1.70	1.47	1.28, 1.68	1.43	1.24, 1.65	1.28	1.03, 1.58
	Provider-initiated	128/1585	8.08	1.33	1.12, 1.57	1.29	1.09, 1.52	1.23	1.02, 1.47	1.14	0.87, 1.48
Spontaneous PTB	Not Preterm	3164/75,172	4.21	1.00	Reference	1.00	Reference	1.00	Reference	1.00	Reference
	Spontaneous	149/2165	6.88	1.64	1.40, 1.92	1.61	1.38, 1.89	1.57	1.32, 1.85	1.33	1.04, 1.71
	Provider-initiated	93/1585	5.87	1.39	1.14, 1.70	1.36	1.11, 1.66	1.32	1.07, 1.64	1.09	0.78, 1.52
Provider-initiated PTB	Not Preterm	1401/75,172	1.86	1.00	Reference	1.00	Reference	1.00	Reference	1.00	Reference
	Spontaneous	46/2165	2.12	1.14	0.85, 1.52	1.13	0.85, 1.52	1.11	0.82, 1.51	1.16	0.76, 1.77
	Provider-initiated	35/1585	2.21	1.18	0.85, 1.65	1.13	0.81, 1.57	1.00	0.69–1.46	1.24	0.77, 1.99

Abbreviations: *PTB* preterm birth, *URR* Unadjusted Relative Risk, *ARR* Adjusted Relative Risk, *CI* confidence intervals

^a^Analyzed with a log-binomial regression model. 276 children excluded due to missing information on maternal PTB type

^b^Adjusted for whether mother moved or had a substance abuse diagnosis in year before birth of child, child’s birth year, mother’s age at birth of child, neighborhood location and SES at birth of child, child’s sex and child’s birth order

^c^Restricted to births 2000–2016 with complete information on additional covariates. Adjusted for whether mother moved or had a substance abuse diagnosis in year before birth of child, child’s birth year, mother’s age at birth of child, neighborhood location and SES at birth of child, child’s sex, child’s birth order, mother’s smoking status during pregnancy, mother’s education, inadequate prenatal care, receipt of employment income assistance in the year before the birth of the child, and whether mother had a partner

^d^Restricted to one randomly child per mother (1995–2016). Adjusted for whether mother moved or had a substance abuse diagnosis in year before birth of child, child’s birth year, mother’s age at birth of child, neighborhood location and SES at birth of child, child’s sex and child’s birth order

In the siblings’ analysis (Table 4) there was no statistically significant intergenerational recurrence patterns, with the possible exception of spontaneous PTB. Mothers of spontaneous PTB status were more likely to deliver spontaneous PTB infants [ARR: 1.39; 95% CI: 0.99, 1.93]. This borderline association barely changed when controlling for additional covariates but weakened after accounting for the recurrence of PTB within births of the same women [ARR: 1.26; 95% CI: 0.73, 2.20].

**Table 4:** Association between maternal and offspring preterm birth type in the sibling subcohort^a^, Manitoba, Canada, 1980–2016

		Infant’s PTB cases/No of births	PTB rate, %	(*N* = 2494)		(*N* = 2494)		(*N* = 2157)		(*N* = 1031)	
Offspring outcome	Maternal PTB type			URR	95% CI	ARR^b^	95% CI	ARR^c^	95% CI	ARR ^d^	95% CI
Any PTB	Not Preterm	143/1425	10.04	1.00	Reference	1.00	Reference	1.00	Reference	1.00	Reference
	Spontaneous	72/611	11.78	1.17	0.85, 1.63	1.18	0.86, 1.63	1.16	0.82, 1.61	1.01	0.64, 1.59
	Provider-initiated	37/458	8.08	0.81	0.54, 1.21	0.80	0.54, 1.17	0.64	0.40, 1.05	0.68	0.38, 1.24
Spontaneous PTB	Not Preterm	90/1425	6.32	1.00	Reference	1.00	Reference	1.00	Reference	1.00	Reference
	Spontaneous	52/611	8.51	1.35	0.97, 1.87	1.39	0.99, 1.93	1.37	0.91, 2.07	1.26	0.73, 2.20
	Provider-initiated	23/458	5.02	0.80	0.51, 1.24	0.78	0.50, 1.20	0.79	0.45, 1.40	0.85	0.42, 1.74
Provider-initiated PTB	Not Preterm	53/1425	3.72	1.00	Reference	1.00	Reference	1.00	Reference	1.00	Reference
	Spontaneous	20/611	3.27	0.88	0.51, 1.51	0.87	0.50, 1.52	0.83	0.49, 1.44	0.65	0.29, 1.46
	Provider-initiated	14/458	3.06	0.82	0.44, 1.54	0.80	0.43, 1.50	e		e	

Abbreviations: *PTB* preterm birth, *URR* Unadjusted Relative Risk, *ARR* Adjusted Relative Risk, *CI* confidence intervals

^a^Analyzed with a generalized estimating equations fixed-effects log-binomial regression model. 5 children excluded due to missing information on maternal preterm type

^b^Adjusted for whether mother moved or had a substance abuse diagnosis in year before birth of child, child’s birth year, mother’s age at birth of child, neighborhood location and SES at birth of child, child’s sex and child’s birth order

^c^Restricted to births 2000–2016 with complete information on additional covariates. Adjusted for whether mother moved or had a substance abuse diagnosis in year before birth of child, child’s birth year, mother’s age at birth of child, neighborhood location and SES at birth of child, child’s sex, child’s birth order, mother’s smoking status during pregnancy, mother’s education, inadequate prenatal care, receipt of employment income assistance in the year before the birth of the child, and whether mother had a partner

^d^Restricted to one randomly birth per mother (1995–2016). Adjusted for whether mother moved or had a substance abuse diagnosis in year before birth of child, child’s birth year, mother’s age at birth of child, neighborhood location and SES at birth of child, child’s sex and child’s birth order

^e^Suppressed due to small number of events (≤6)

## Discussion

We found that maternal preterm birth status was positively associated with offspring preterm birth in the general population of Manitoba but not in a subcohort of sisters with discordant preterm birth status who subsequently gave birth. In the sister subcohort, preterm delivery was equally elevated among women born preterm themselves and among those who were born at term but had a sister who was born preterm. Observed intergenerational patterns were more apparent for spontaneous PTB.

Our findings for the population cohort align remarkably well with those of previous studies. In the population cohort we obtained an unadjusted RR: 1.42 (1.27, 1.58), similar to that of a meta-analysis involving seven studies with low heterogeneity [RR: 1.41 (1.26, 1.59)] [9]. Like others, we also found a dose-response association with mothers born at earlier gestation in the population cohort [15]. However, the apparent lack of association in our sibling analyses indicates that the higher risk observed among women born preterm was shared by their sisters not born preterm, suggesting that the actual risk factor for preterm birth may not be maternal preterm birth status per se but a broader familial occurrence of preterm birth. Two studies using different designs also found a higher risk of delivering preterm shared by sisters of concordant or discordant PTB status but not by brothers [30, 31], which suggests that paternal and fetal genes may not play an important role and that the transmission of PTB mainly occurs through the female line [15, 30, 31].

We also observed that sisters of concordant PTB status exhibited extreme outcomes. PTB rates were lowest among sisters all born at term (6.1%) and highest among sisters all born preterm (13.1%). These findings are consistent with polygenic theory according to which higher recurrence rates occur among close relatives if more than one family member is affected [32]. Although our study points at familial female aggregation of PTB, it cannot specify the contributions of genetic and environmental factors involved, since the sibling design keeps these characteristics shared by siblings constant [29]. Studies on the heritability of preterm birth have shown that fetal and maternal genetic components play a role, although secondary to that of environmental factors [13, 14]. Further studies may elucidate the differential contribution of genetic and familial environmental influences.

In our analyses stratified by preterm birth types, there was no intergenerational association for infant provider-initiated PTB in the population cohort or in the sibling cohort. Infant spontaneous PTB exhibited clear associations with maternal spontaneous PTB in the population cohort across all models. In the sibling analysis, these associations were positive but not statistically significant presumably due to reduced statistical power to detect associations. Given this limitation, the possibility of a true maternal transmission of spontaneous PTB cannot be ruled out. Further studies may clarify this issue.

Our study has limitations. The use of administrative data collected for other purposes restricted the information available for analyses. At the same time, routine data collection on a relatively stable and large population over decades made this study possible. Our sibling design is particularly suited to draw causal inferences regarding an individual exposure that differs between siblings, provided that there is no exposure measurement error or confounding due to factors not shared by siblings [33]. Gestational age information was lacking for 19% of pregnancies, most of them affecting maternal birth status. A validation study indicated that PTB (< 37 weeks) in Canadian hospital records has sensitivity and specificity of 91.2 and 98.8%, respectively [23]. Measurement of gestational age is known to vary over time, being more likely to be influenced by the last menstrual period (LMP) when sister mothers were born than when they delivered. LMP may overestimate PTB rates [34], which would artificially increase maternal exposure to PTB and dilute associations, particularly in the siblings’ analyses [33]. However, in our data very and moderate PTB rates were not higher in the 1980’s and 1990’s, when mothers were born, and secular trends are consistent with those reported in North America [35]. Despite controlling by design for genetic and environmental factors shared by siblings, unmeasured individual risk factors not shared by sister mothers may contribute to some degree of residual confounding. Our sensitivity analysis adjusting for additional maternal risk factors (e.g., maternal smoking, education, prenatal care, no partner, receipt of income assistance) suggests that such bias would be small since estimates remained virtually unchanged. Previous studies have reported a similar low impact of covariate control [5–8, 31]. We did not control for race or ethnicity, which may be warranted in a multiethnic population characterized by immigration and a large share of Indigenous people, who experience social and health disadvantage [36]. However, unlike in the population analyses, race/ethnicity is controlled for by the sibling design, which in turn partially controls for other characteristics correlated to race/ethnicity and reduces their variability between siblings. We did not control for maternal morbidity, which may be in the causal pathway. We could not include stillbirths, many of whom are preterm, because only live births receive a birth record linkable to the mother. Underestimation of preterm birth rates would be small due to the rare occurrence of stillbirths. Finally, we did not have usable data on fathers or partners who may have helped determine if women in the sibling cohort were full or half sisters. Paternal factors may also influence pregnancy courses, although not through their genes [15].

Our findings provide new insights into the study of intergenerational effects of PTB. First, our study provides additional evidence that PTB clusters within families and that maternal preterm birth status may not be a predictor of preterm delivery as sensitive as a family history of PTB. Interestingly, the risk of offspring PTB within families in which at least one mother-sister was born preterm was higher (≥10%) than among women born preterm without sisters (7.7%). Part of this excess risk may be due to poorer sociodemographic profiles aggravated by larger family size, so that the environmental factors contributing to PTB among women born preterm seem to affect equally their sisters not born preterm themselves. The association between socioeconomic disadvantage and adverse birth outcomes is well established not only cross-sectionally [12] but also across generations [37]. The clustering of PTB risk within families may also be influenced by gene-gene and gene-environment interactions. Anthropometric features, such as maternal height and body size, are also inherited to some extent and associated with PTB [38, 39]. The heritability of PTB has been estimated in the range of 15 to 40% [17], although an unknown part of the recurrence across generations may involve non-genetic factors [15].

Second, both the population and the sibling analyses indicated that provider-initiated PTB was not influenced by maternal or familial PTB. The possibility that the event of being born preterm, irrespective of the cause, may physiologically predispose women to deliver preterm later in life (i.e., adverse fetal programming) is not supported by our findings. A focus on spontaneous PTB may better serve to identify mechanisms involved in familial patterns of PTB.

## Conclusions

Our findings do not support the notion of seeing maternal PTB status per se as an individual risk factor for preterm delivery. Rather, consideration of a female family history of PTB may better identify women at higher risk of preterm delivery and perhaps other pregnancy complications. The clustering of PTB within families suggests that family-based approaches to understanding the occurrence of preterm birth and its sequelae may be more fruitful than solely focusing on individual risk factors.

## Abbreviations

ARR: Adjusted Relative Risk
CI: Confidence Interval
ICD: International Classification of Diseases
LMP: Last Menstrual Period
PTB: preterm birth
RR: Relative Risk
